# Supplementary material for: Active learning of reactive Bayesian force fields applied to heterogeneous catalysis dynamics of H/Pt
Source: Nat Commun. 2022 Sep 2;13:5183. doi: 10.1038/s41467-022-32294-0 (PMC9440250; doi:10.1038/s41467-022-32294-0)
Supplement: Supplementary file 1 — Supplementary Information [file 41467_2022_32294_MOESM1_ESM.pdf]

Supplementary Information

Active learning of reactive Bayesian force fields applied to heterogeneous catalysis dynamics of H/Pt

J. Vandermause et al.

SUPPLEMENTARY FIGURES

Additional data on training & simulation

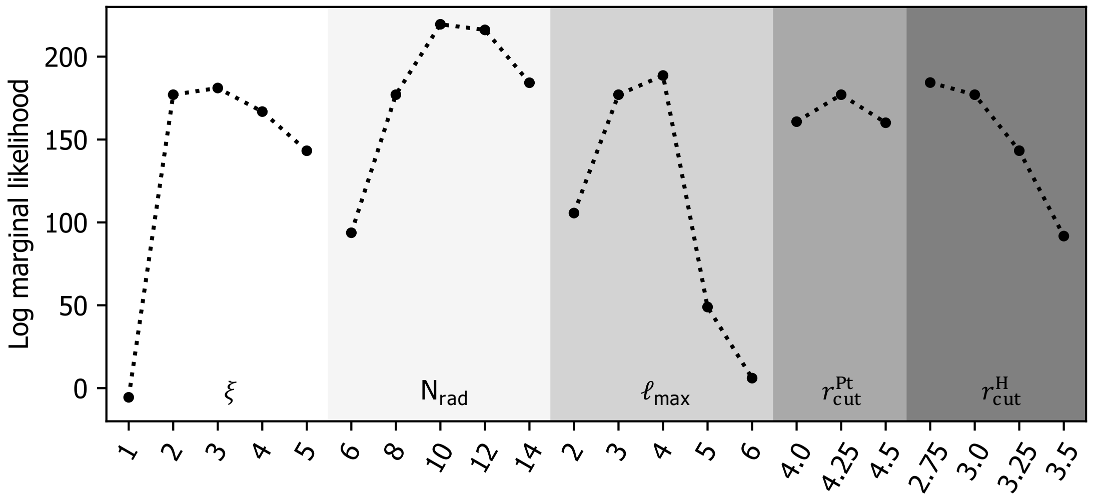

Supplementary Figure 1. The SGP likelihood for various integer kernel powers ξ, basis set expansion parameters N_{rad} and ℓ_{max}, and model cutoffs $r_{\mathrm{cut}}^{\mathrm{Pt}}$ (denoting the Pt-Pt cutoff) and $r_{\mathrm{cut}}^{\mathrm{H}}$ (denoting the Pt-H and H-H cutoffs, set equal to each other). The likelihood was evaluated with five structures in the training set of the SGP from the H/Pt(111) training simulation.

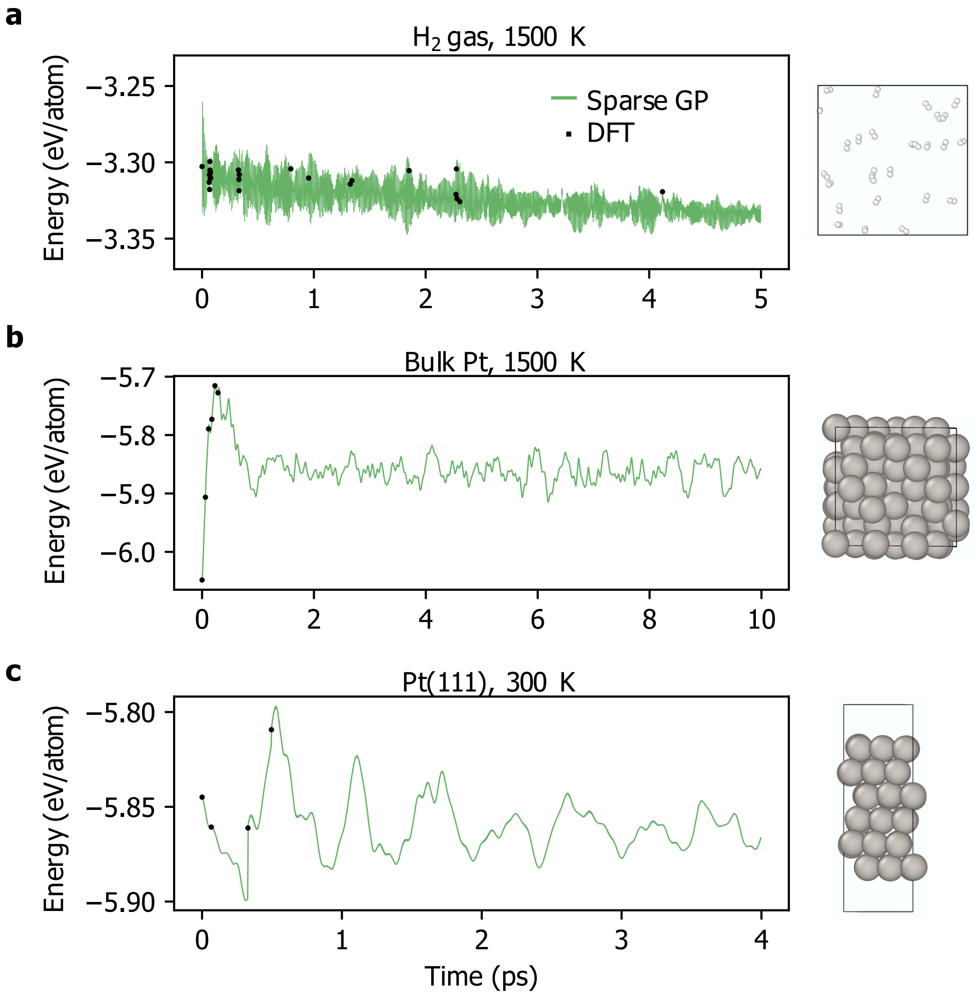

Supplementary Figure 2. Potential energy versus time for the on-the-fly training simulations reported in the main text: (a) H_2 gas at 1500 K; (b) bulk Pt at 1500 K; and (c) a six-layer slab model of Pt(111) at 300 K. Example structures from the simulations are shown on the right.

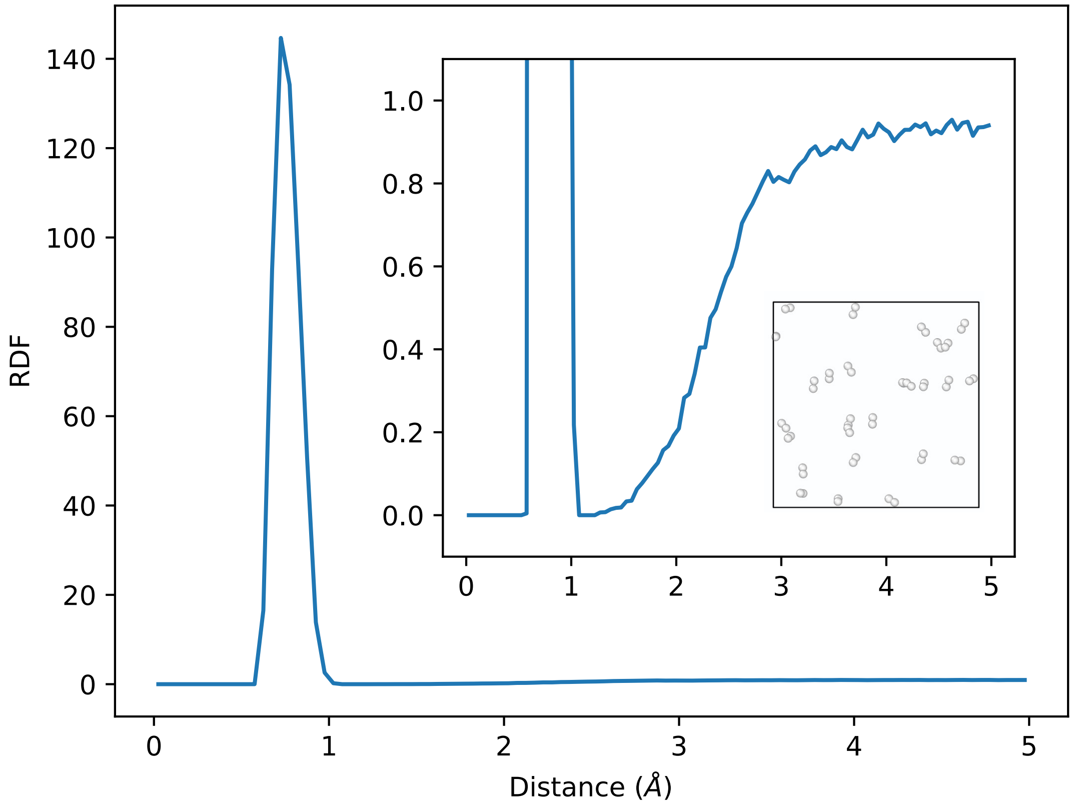

Supplementary Figure 3. Time-averaged radial distribution function of the H_2 training simulation.

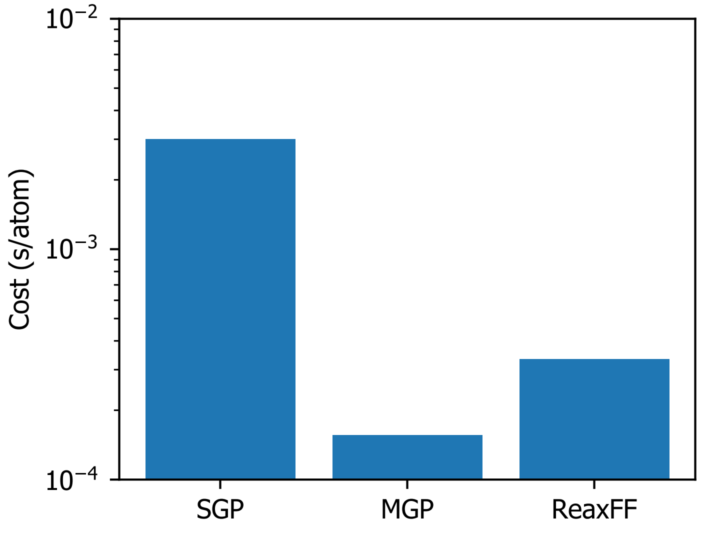

Supplementary Figure 4. Prediction cost of the SGP, the equivalent mapped model implemented in LAMMPS (labeled MGP), and the H/Pt ReaxFF model [1]. A single CPU was used to evaluate the models on the same structure, which consisted of a single H atom adsorbed on a six-layer slab model of a 3×3 unit cell of Pt(111).

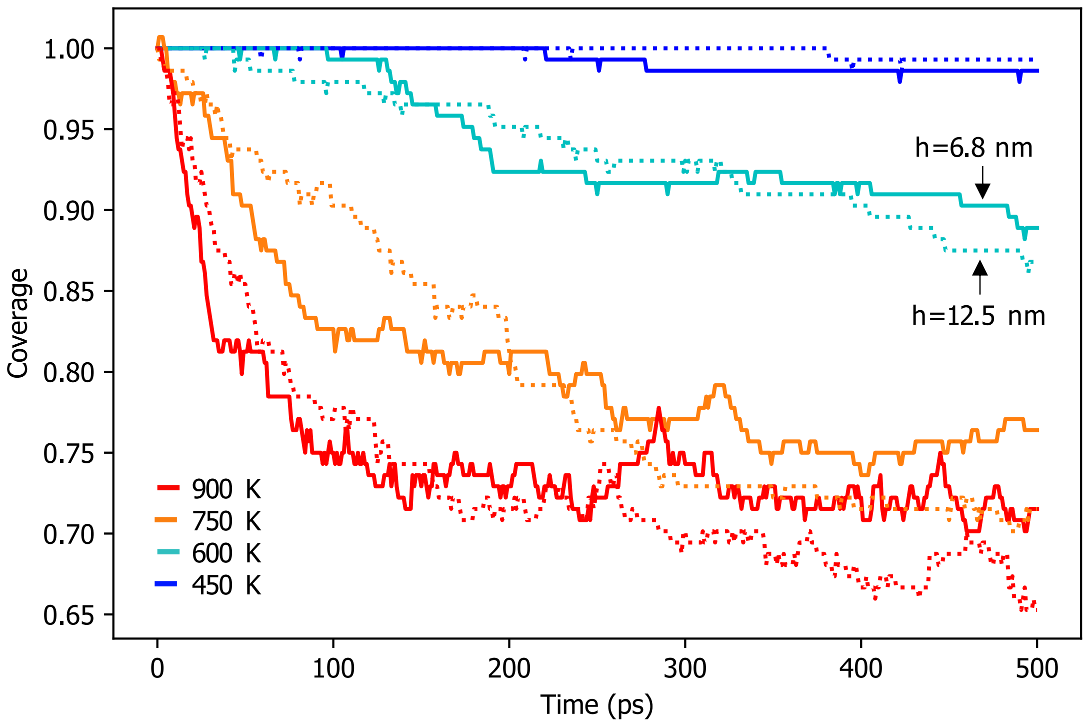

Supplementary Figure 5. Surface H coverage versus time for the large-scale MD simulations. Two box heights are considered: 6.8 nm (solid) and 12.5 nm (dotted).

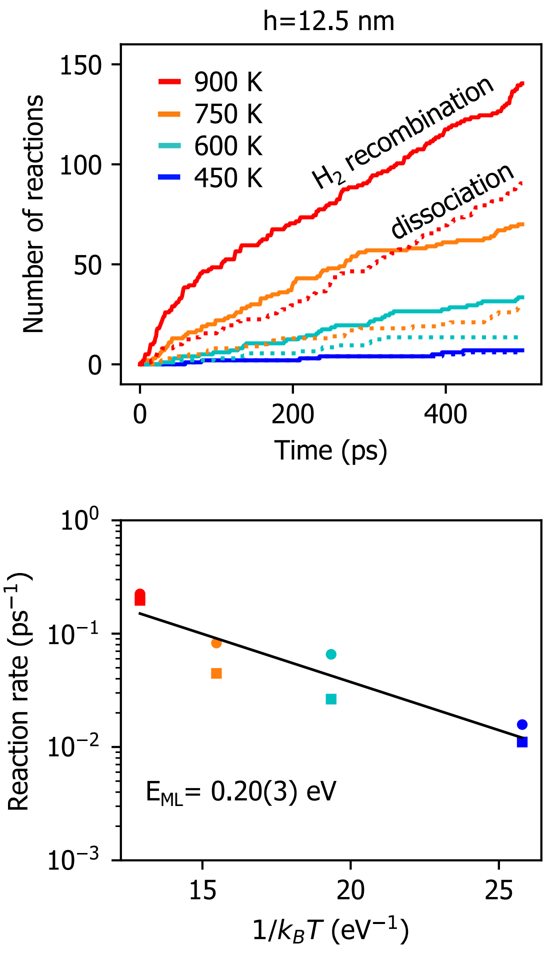

Supplementary Figure 6. Cumulative number of H_2 reactive events (top) and the corresponding Arrhenius plot (bottom) for the simulations conducted with the box height of 12.5 nm.

Mean absolute errors, parity plots, & comparisons with ReaxFF

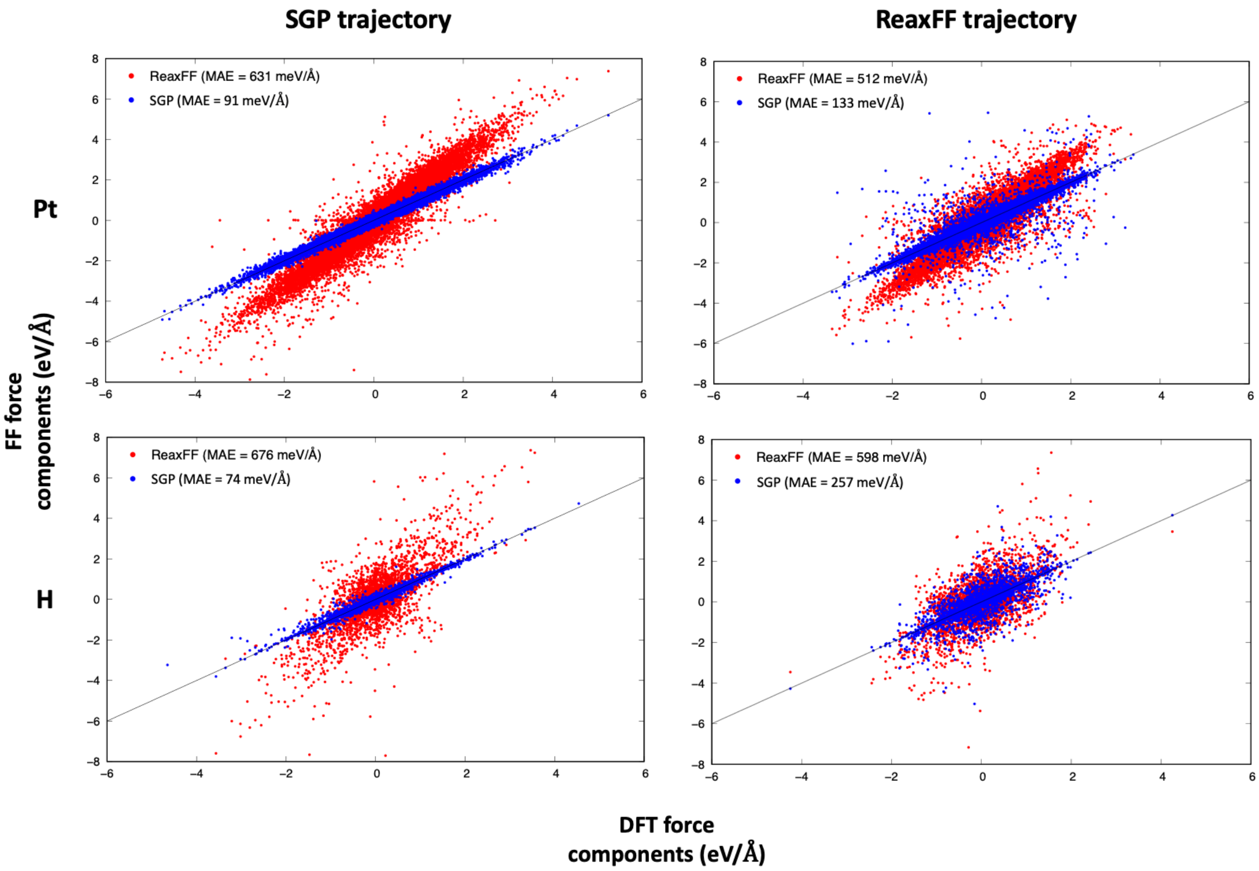

Supplementary Figure 7. Parity plot of the force components predicted by the force field (FF) vs. DFT for Pt and H (top and bottom), evaluated on the SGP and ReaxFF trajectories (left and right). The parity lines are shown in black. Predictions of the SGP model and the ReaxFF model [1] are shown in blue and red, respectively. The mean absolute error (MAE) values are indicated in the legend (see Supplementary Table 1).

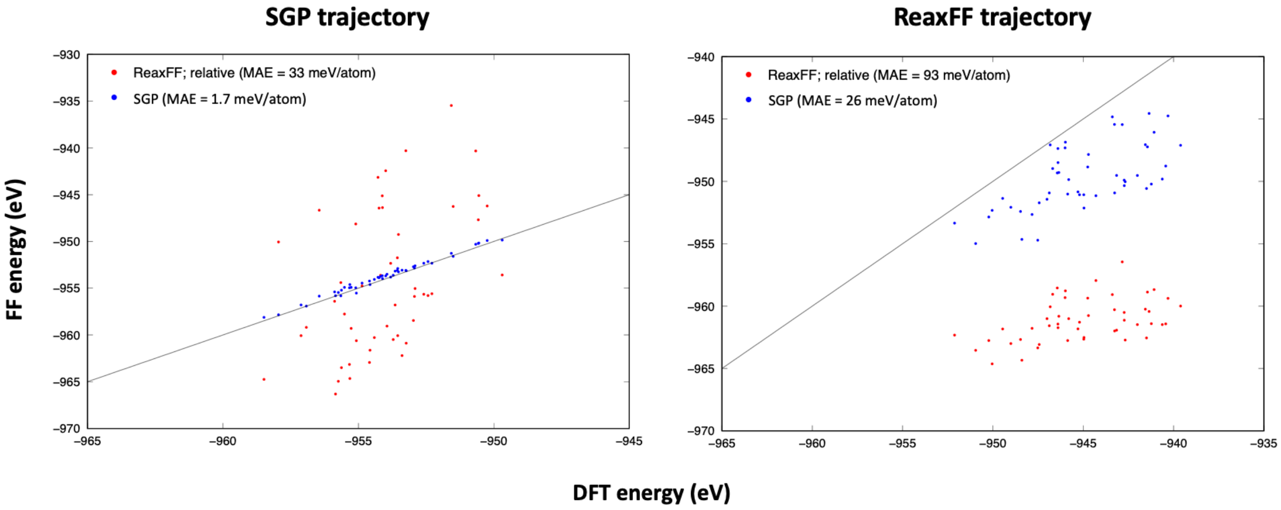

Supplementary Figure 8. Parity plot of the potential energies predicted by the force field (FF) vs. DFT, evaluated on the SGP and ReaxFF trajectories (left and right). The parity lines are shown in black. Predictions of the SGP model and the ReaxFF model [1] are shown in blue and red, respectively. The mean absolute error (MAE) values are indicated in the legend (see Supplementary Table 1). To align the reference, the ReaxFF energy values were shifted by the DFT energy value of the initial relaxed structure.

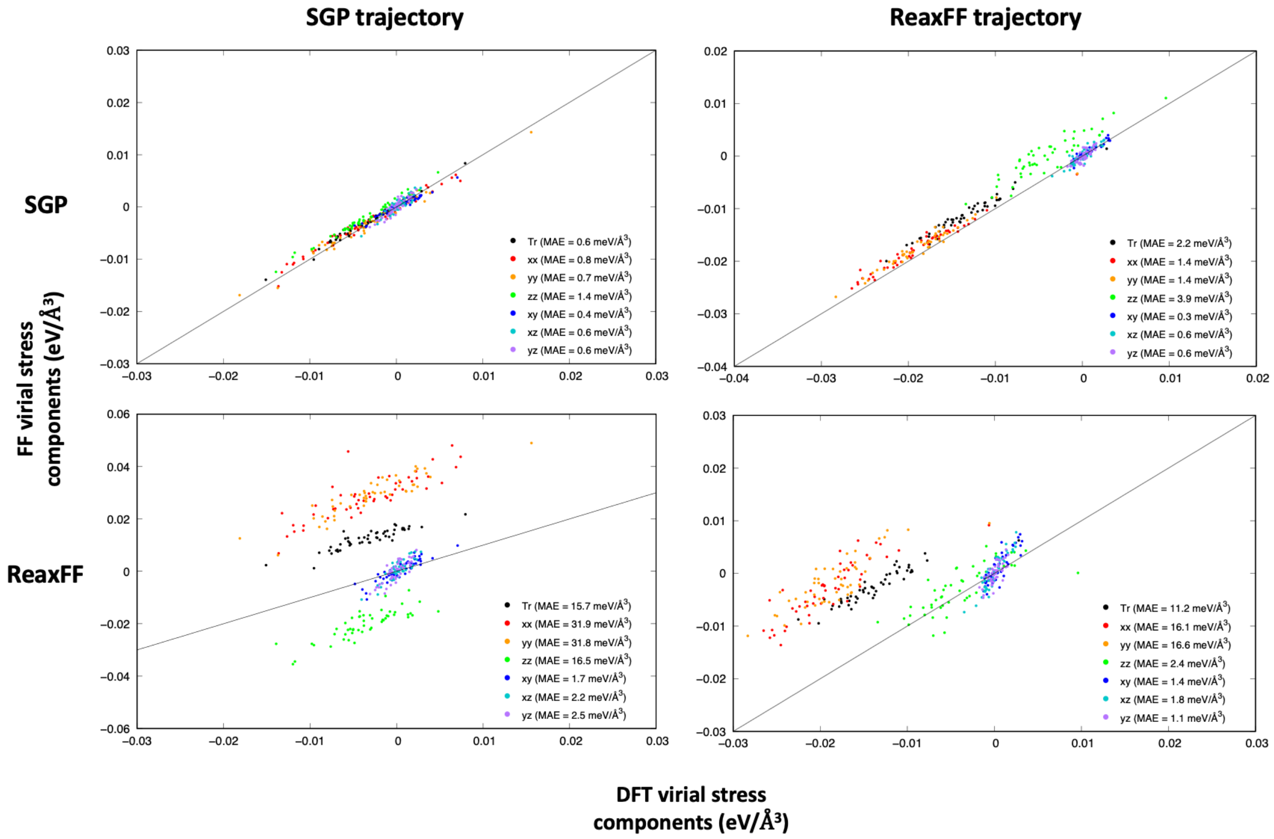

Supplementary Figure 9. Parity plot of the virial stress components predicted by the force field (FF) vs. DFT, evaluated on the SGP and ReaxFF trajectories (left and right). The parity lines are shown in black. Predictions of the SGP model and the ReaxFF model [1] are shown in top and bottom, respectively. The six components and the trace are labeled by different colors, with the corresponding mean absolute error (MAE) values indicated in the legend (see Supplementary Table 1).

Transition state pathways

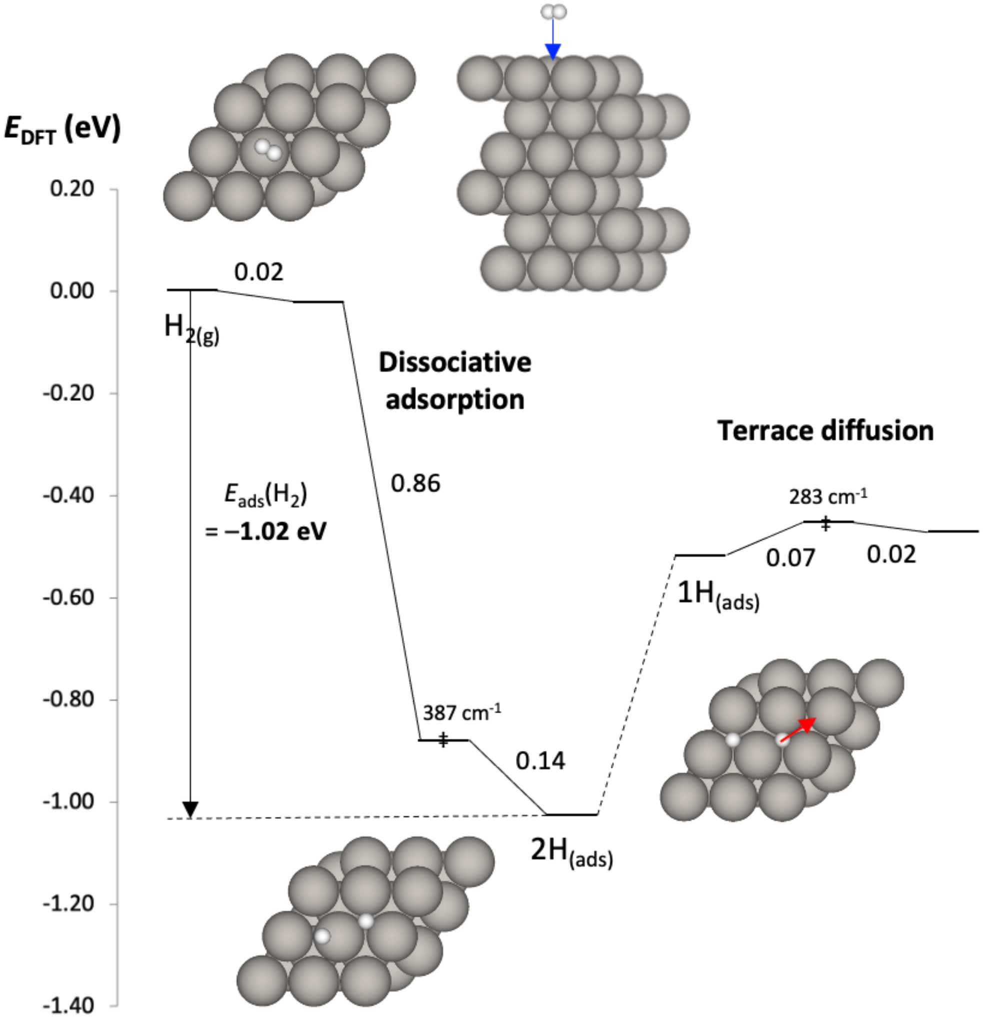

Supplementary Figure 10. Minimum energy pathway for H_2 dissociative adsorption and atomic H diffusion on Pt(111), optimized using DFT. Numbers indicate energy differences between adjacent states. Transition states are labeled with ‡, above which the corresponding imaginary frequency is shown. The dotted line indicates a change of reference from $H_{2(g)}$ to $\frac{1}{2}H_{2(g)}$ for $1H_{(ads)}$ states.

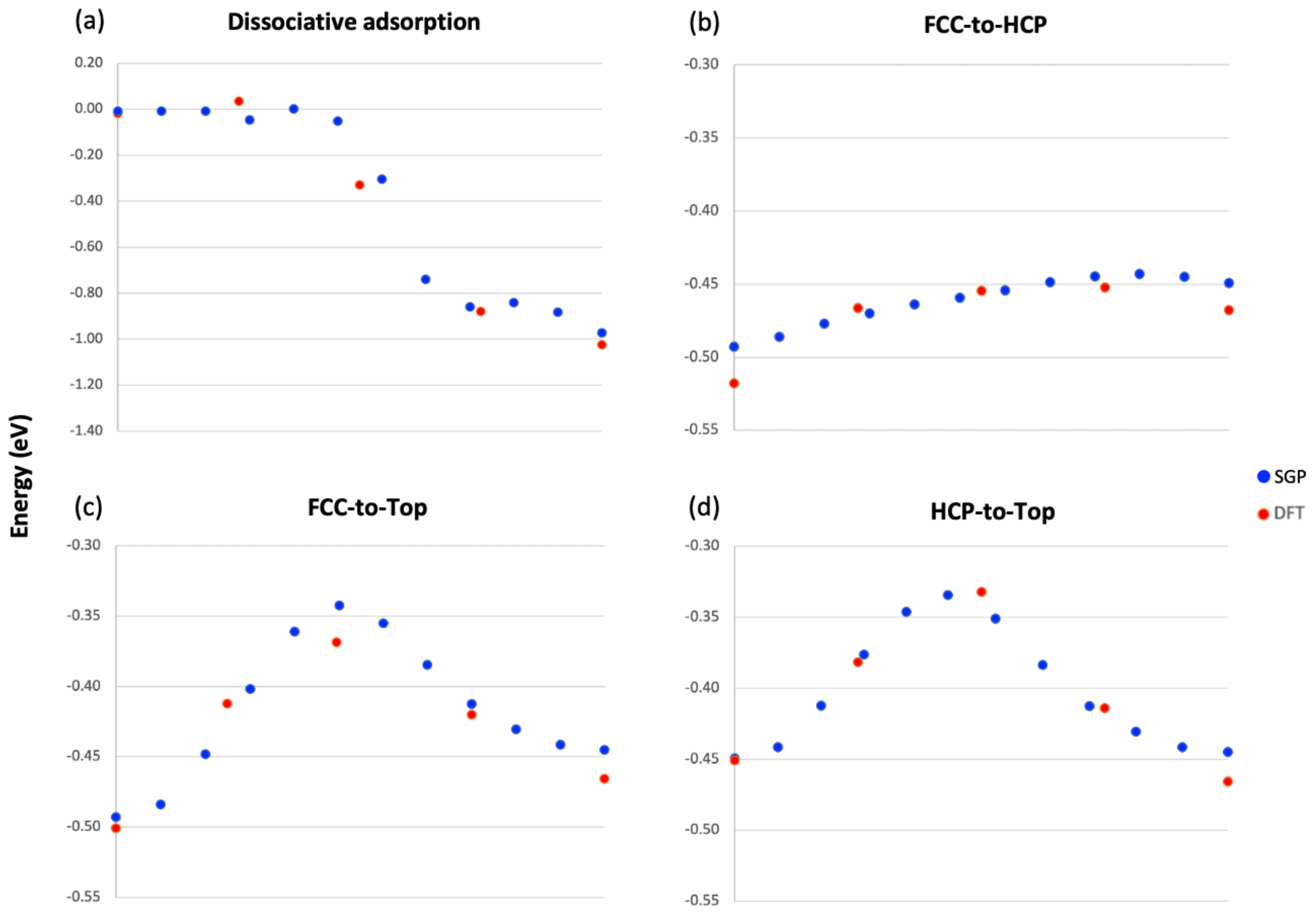

Supplementary Figure 11. Transition state pathways for (a) H_2 dissociative adsorption; and atomic H diffusion on Pt(111): (b) FCC to HCP hollow; (c) FCC hollow to atop; (d) HCP hollow to atop. In all cases, our SGP model (blue) is in good agreement with DFT (red). The references for the chemisorption and diffusion processes are $H_{2(g)}$ and $\frac{1}{2}H_{2(g)}$, respectively.

Arrhenius analysis of atomic H diffusion

We simulate atomic H diffusion on Pt(111) with our SGP model at 300-900 K for 200 ps. For the starting structure, we use a 10×10 unit cell of Pt(111) with one H atom placed at the FCC hollow site. As shown in Supplementary Figure 12, linear surface diffusion is maintained up to ∼900 K, above which nontrivial vertical motion of the H atom is observed, either desorption or subsurface diffusion, as seen in e.g. large fluctuation of the z-component of the MSD at 1200 K.

The diffusion coefficient is defined as

$$D = \lim_{t \to \infty} \frac{\langle [r(t) - r(0)]^2 \rangle}{2dt}, \tag{1}$$

where r is the adatom position, time zero is set to the beginning of the linear diffusive regime, and $d = 3$ is the dimensionality of the system. The MSD is ensemble-averaged over 30 independent simulations to ensure sufficient noise reduction. Temperature dependence of the diffusion coefficient shows an Arrhenius behavior (Supplementary Figure 13),

$$\ln(D) = \ln(D_0) - \frac{E_\mathrm{a}}{k_\mathrm{B}T}, \tag{2}$$

from which the corresponding activation energy (E_a) and diffusion prefactor (D_0) are obtained as 92 meV and 3.53×10^{13} Å^2/s, respectively. As the temperature range of 300-900 K corresponds to an average thermal energy of $k_\mathrm{B}T$ = 26-78 meV, which lies below the obtained barrier of 92 meV, the simulated diffusion remains a thermally activated process.

Using the exact same starting structure—a six-layer slab model of a 12×12 unit cell of Pt(111) with 1 ML H coverage on both sides of the slab and 80 randomly oriented H_2 molecules in the gas phase—we also simulate H_2 reactivity using the ReaxFF model [1] at the same temperature range of 300-900 K. As shown in Supplementary Figure 14, ReaxFF leads to facile surface evaporation of isolated Pt hydride units at 600 K and above, ultimately resulting in surface vacancy clusters. As such, the Arrhenius analysis was not performed for this model.

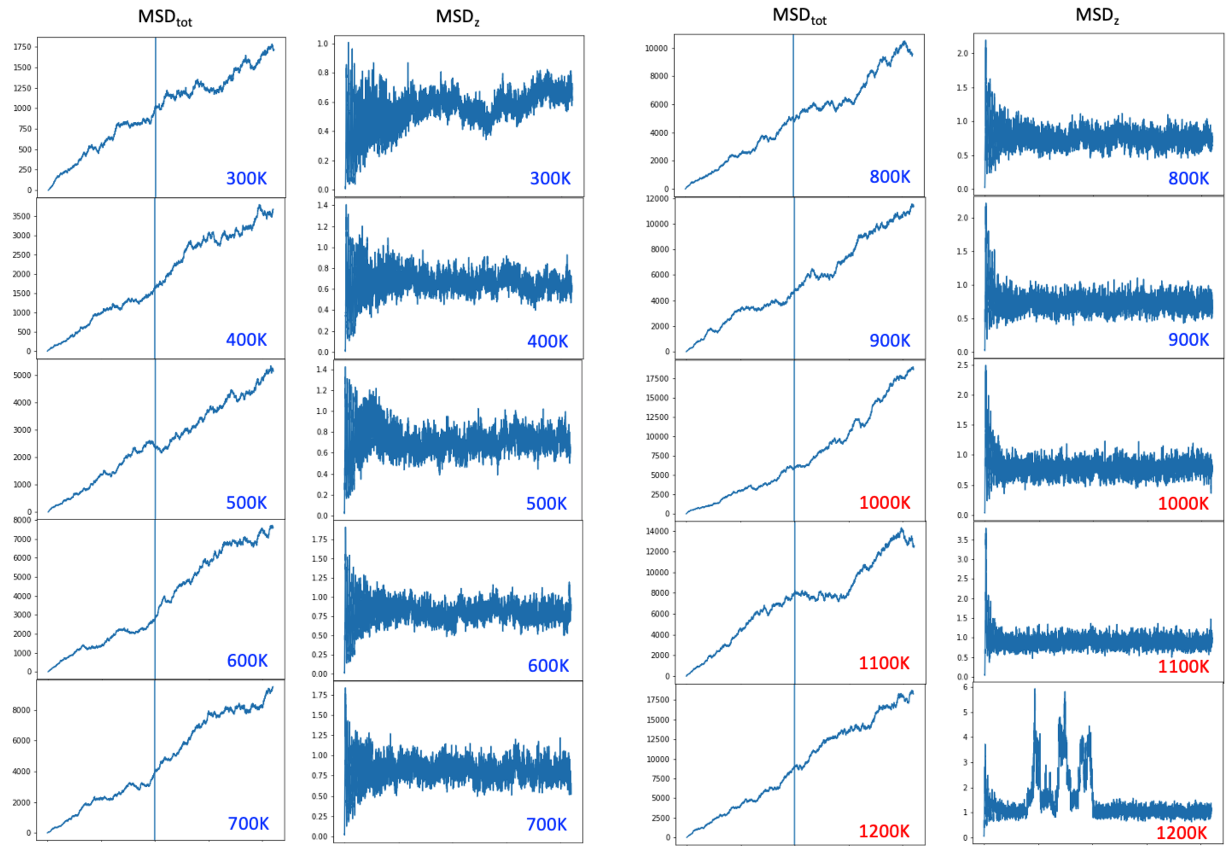

Supplementary Figure 12. Plots of ensemble-averaged mean-squared displacement (MSD) (Å^2) of H atom on Pt(111) over 200 ps at 300-1200 K, showing the total MSD ($\mathrm{MSD_{tot}}$) and the corresponding z-component (MSD_z). The second half of the simulations (half-time indicated by vertical lines) are taken as linear diffusive regimes. Above 900 K, nontrivial vertical motion of the H atom is detected.

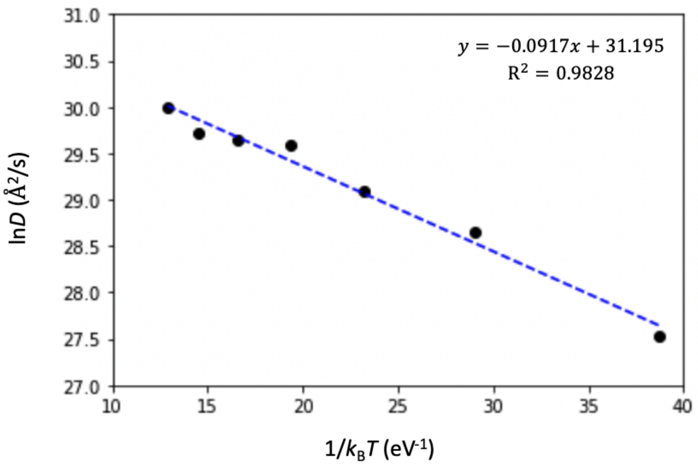

Supplementary Figure 13. Arrhenius plot of simulated atomic H diffusion on Pt(111) at 300-900 K.

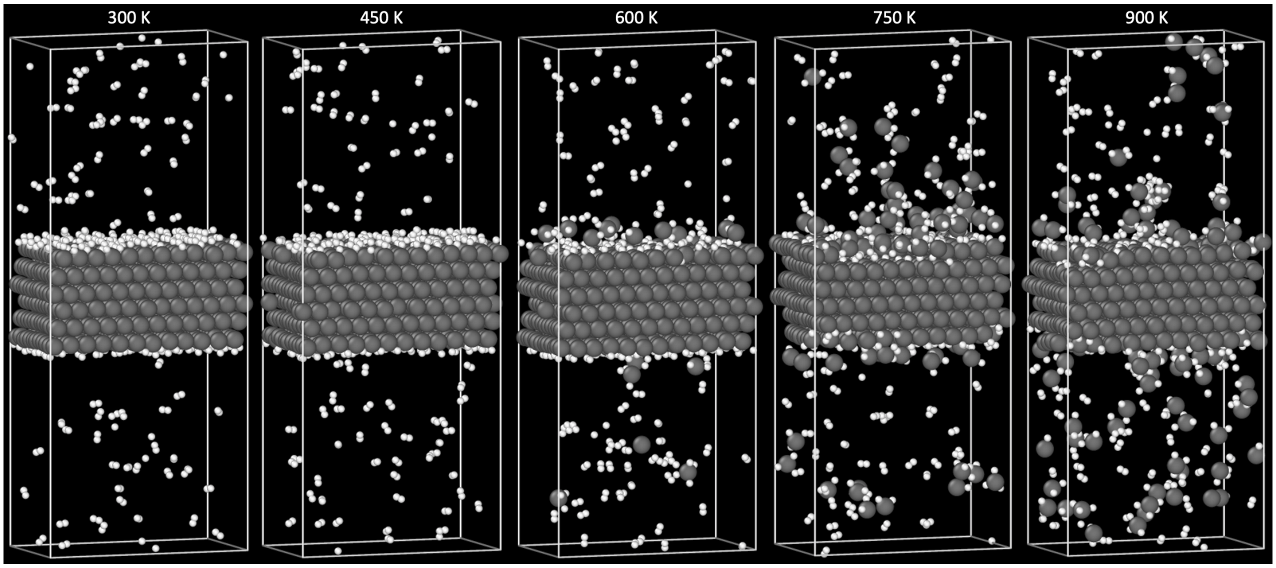

Supplementary Figure 14. Final snapshots of 500 ps simulations of H_2 reactivity on Pt(111) at 300-900 K, using the ReaxFF model [1]. At 600 K and above, the surface undergoes facile evaporation of gaseous Pt hydride (see Sec. 2 for validation against DFT).

Performance and scaling

In this section, we present performance data quantifying the prediction speed of our SGP and mapped SGP LAMMPS models. The data set used for these tests consists of 200 DFT frames obtained from the H/Pt training simulation described in the main text. To sweep the training set size, we add energies, forces, and stresses of up to 200 frames to the SGP training set, with 10 sparse environments added to the sparse set for each frame. To sweep the size of the descriptor vector, we fix $l_{max} = 3$ and vary n_{max} between 2 and 16 in increments of 2. The corresponding descriptor dimension n_{desc} ranges from 40 to 2112 for the two-species H/Pt models (see Eq. (7) of the main text). Both the SGP and LAMMPS tests are performed on a single CPU without parallelization.

Supplementary Figure 15 plots prediction times of SGP and mapped SGP LAMMPS models as a function of the descriptor dimension n_{desc}. To probe the species-dependence of the prediction time of the LAMMPS models, we include in the comparison a one-species model obtained by treating H and Pt atoms as identical species (yellow dashed line in Supplementary Figure 15) and a three-species model obtained by introducing a "dummy" species that increases the size of the descriptor vector according to Eq. (7) of the main text (pink dashed line). For the LAMMPS models, quadratic growth in the prediction cost is observed for $n_{\text{desc}} \gtrsim 500$, consistent with the quadratic form of the energy model.

In Supplementary Figure 16, we report the fraction of the total prediction time spent computing descriptors. For SGP models with a sufficiently large number of sparse environments ($n_{\text{sparse}} \geq 810$), the majority of the prediction time consists of kernel evaluations rather than descriptor evaluations. For the mapped SGP model, because energy evaluations are quadratic in the descriptor, the fraction of time spent computing descriptors drops to zero as the descriptor dimension increases (solid dashed line in Supplementary Figure 16).

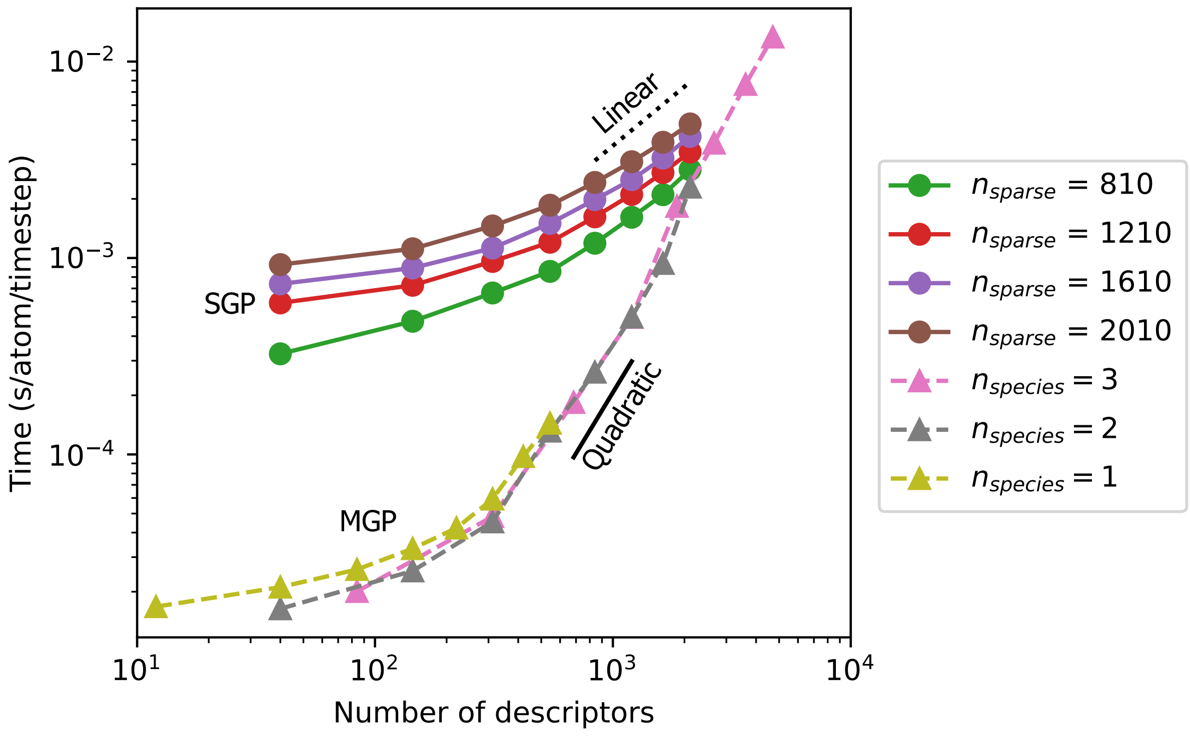

Supplementary Figure 15. Scaling of SGP (solid line) and mapped SGP (dashed line) prediction times with the descriptor dimension. For the mapped SGP models, we include one- and three-species models (yellow and pink, respectively). Linear and quadratic scaling are shown as dotted and solid black lines, respectively.

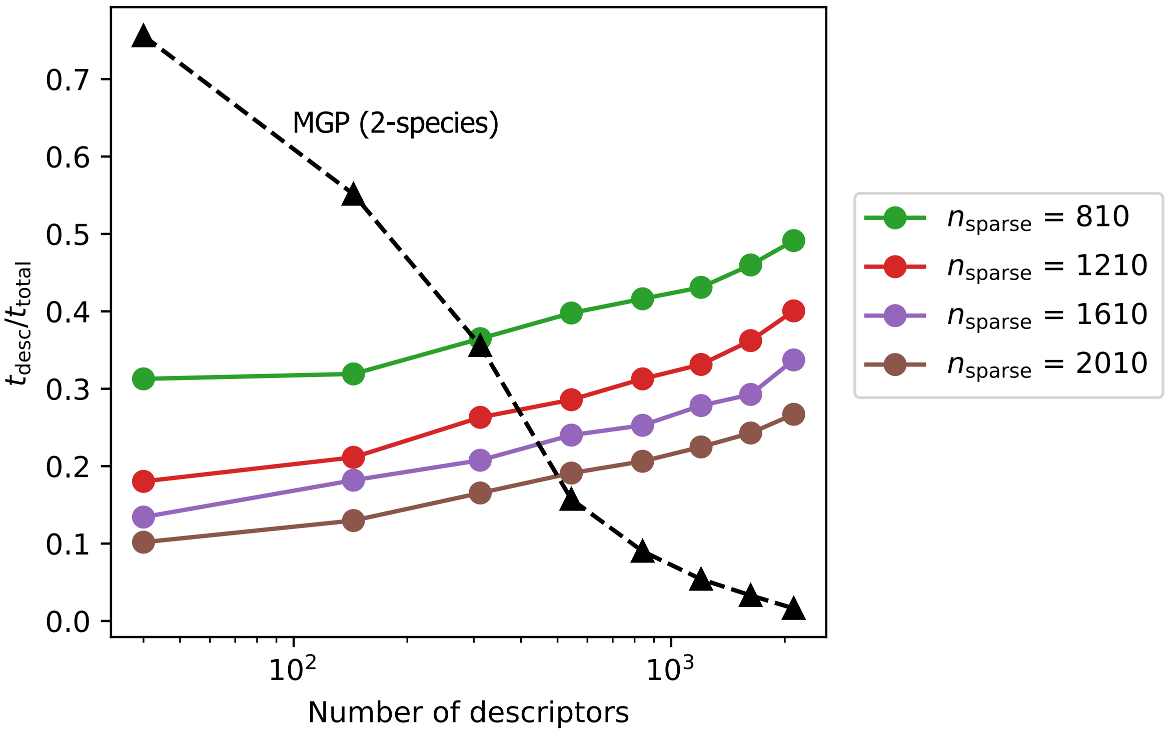

Supplementary Figure 16. Fraction of the total prediction time spent computing descriptors.

SUPPLEMENTARY TABLES

Mean absolute errors (MAE) of the predictions of the SGP model and the ReaxFF model [1] with respect to DFT are presented in Supplementary Table I for three major properties: (i) atomic force components of Pt and H; (ii) total potential energy; and (iii) virial stress tensor components, including the trace. The predictions are made on 500-ps trajectories generated independently by both models. The MAE values are colored by high (red), medium (orange), and low (green). See Supplementary Figures 7-9 for the corresponding parity plots.

Supplementary Table I. MAEs of forces, energies, and stresses.

Property	Force field	Component	SGP trajectory	ReaxFF trajectory
Forces (meV/Å)	SGP	Pt	91	133
		H	74	257
	ReaxFF	Pt	631	512
		H	676	598
Energy (meV/atom)	SGP	Total	1.7	26
	ReaxFF	Total	33	93
Virial stress (meV/Å^3)	SGP	Tr	0.6	2.2
		xx	0.8	1.4
		yy	0.7	1.4
		zz	1.4	3.9
		xy	0.4	0.3
		yz	0.6	0.6
		xz	0.6	0.6
	ReaxFF	Tr	15.7	11.2
		xx	31.9	16.1
		yy	31.8	16.6
		zz	16.5	2.4
		xy	1.7	1.4
		yz	2.2	1.8
		xz	2.5	1.1

SUPPLEMENTARY REFERENCES

[1] Lili Gai, Yun Kyung Shin, Muralikrishna Raju, Adri CT van Duin, and Sumathy Raman, "Atomistic adsorption of oxygen and hydrogen on platinum catalysts by hybrid grand canonical monte carlo/reactive molecular dynamics," J. Phys. Chem. C **120**, 9780–9793 (2016).
